# Letter to the editor: Multiple large preretinal hemorrhages in the context of severe acute respiratory syndrome coronavirus 2 infection

**DOI:** 10.5935/0004-2749.20210123

**Published:** 2025-08-21

**Authors:** Ana M. Alonso-Tarancón, María R. Sanabria, Santiago García de Arriba, Marina P. González de Frutos, Paola S. Calles-Monar, Saúl Villoria-Díaz

**Affiliations:** 1 Department of Ophthalmology, Complejo Asistencial Universitario de Palencia, Palencia, Spain; 2 Institute of Applied Ophthalmobiology, University of Valladolid, Valladolid, Spain

Dear Editor:

The first cases of coronavirus disease (COVID-19) in Spain were detected in mid-February 2020. One of the highest incidence rates of COVID-19 infection occurred during the first weeks of the pandemic, especially among health-care workers^(1)^.

A recent review about ocular alterations in the context of COVID-19 infection gathered information on a wide range of manifestations from the ocular surface to the posterior segment^(2)^. While most reports refer to ocular surface involvement (at a prevalence rate of 34.5%), retinal manifestations were reported as isolated cases in a cross-sectional study with a small sample^(2)^. Ocular manifestations of COVID-19 are caused by direct damages due to the virus, enhanced by an indirect effect secondary to different processes such as a hypercoagulable environment, immune system response, alterations due to vasoactive pharmacological support, and organ failure^(2)^.

Cell damage due to severe acute respiratory syndrome coronavirus 2 (SARS-CoV-2) includes endothelial cell injury, which has been observed in several organs with histopathological evidence of endothelitis and vasculitis^(3)^. Furthermore, remnants of the COVID-19 RNA were detected in the retina of affected patients^(3)^. Clinical signs of this retinal involvement, such as cotton-wool spots and microbleeding along the vascular arcades and hyperreflective lesions in the ganglion cell and internal plexiform layers, have been found in optic coherence tomography (OCT) assessments of adults examined after recovering from COVID-19^(3)^.

On April 8, 2020, a 54-year-old female nurse with no relevant ophthalmological history visited the emergency department because of the appearance of a large dark spot and pain in her left eye (LE) upon awakening. She did not report headache, trauma, cough, or performing Valsalva maneuver in the previous week. Ten days before the visual loss, she had an episode of fever, cough, asthenia, and general malaise that lasted for 2 days. She had arterial hypertension, which was adequately controlled with treatment.

**Figure 1:**
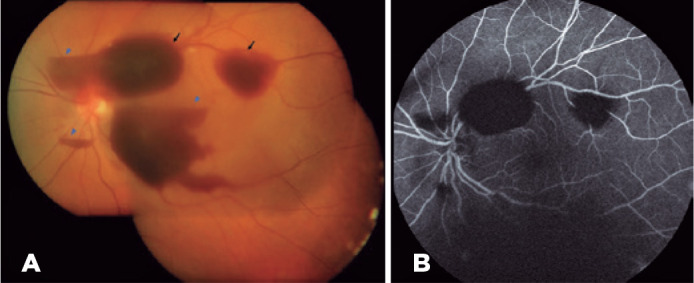
Preretinal hemorrhages of the left eye in the first examination. (A) Real color retinography image showing mild vitreous and large preretinal hemorrhages on the posterior pole, with subhyaloid (blue arrowheads) and sub-internal limiting membrane hemorrhages (black arrowheads). The hemorrhages showed a double-ring sign, with the outer ring representing the subhyaloid hemorrhage and the inner ring representing the sub-internal limiting membrane hemorrhage. (B) The fluorescein angiogram shows the blocking effect of the hemorrhages; no ischemia or vessel alterations can be observed.

Ophthalmic examination revealed a best-corrected visual acuity of 20/125 in the LE, without any changes in the anterior segment. The intraocular pressure was normal. Fundus examination of the LE revealed mild vitreous and multiple large preretinal hemorrhages in the macular area (Figure 1A). The right eye (RE) examination result was normal.

**Figure 2:**
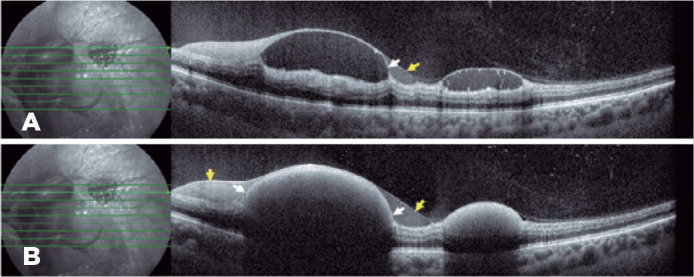
Optical coherence tomography image from the first examination. (A) Horizontal line scan above the level of sedimented blood, exhibiting two distinct membranes, a highly reflective band immediately above the preretinal hemorrhage corresponding to the sub-internal limiting membrane (white arrowhead) and an overlying membrane with low optical reflectivity consistent with the posterior hyaloid (yellow arrowhead). (B) Horizontal line scan at the level of the sedimented blood, showing an intermediate reflectivity line (arrow) corresponding to the inner limiting membrane, splitting the hemorrhage into sub-hyaloid and sub-internal limiting membranes.

OCT (Spectralis Heidelberg Engineering, Heidelberg, Germany) of the LE revealed multiple subhyaloid and sub-internal limiting membrane (ILM) hemorrhages of variable size in the posterior pole and periphery near the vascular arcades (Figure 2). Fluorescein angiography revealed only blocking effect of the hemorrhages (Figure 1B).

Blood analysis revealed mild lymphopenia and an elevated erythrocyte sedimentation rate. Two weeks after the event, the patient tested positive for SARS-CoV-2 IgG antibodies in a chemiluminescence test. Although reverse transcription polymerase chain reaction-based testing is the preferred choice for the laboratory diagnosis of SARS-CoV-2 infection, viral-specific IgM and IgG detection is valid for the serological diagnosis of COVID-19 infection, with high sensitivity and specificity^(4)^. The patient showed a progressive spontaneous resorption, which improved her visual acuity to 20/20 after 8 weeks.

The bleeding complications of SARS-CoV-2 are less known but are also frequent and can affect up to 5% of patients, even those with mild disease. In the present case, the SARS-CoV-2 infection might have played a role in the appearance of the vitreous and retinal hemorrhages.

Subhyaloid and sub-ILM preretinal macular hemorrhages have been described in the context of various illnesses such as diabetic retinopathy, hypertensive retinopathy, retinal artery macroaneurysm, Valsalva retinopathy, Terson’s syndrome, blood dyscrasias, or infectious diseases such as bacterial meningitis and leptospirosis^(5)^. Although retinal complications are rare, given the dimensions of the pandemia, Karampelas et al. emphasized the importance of reporting clinically significant ocular symptoms such as scotomas, especially in patients with diabetes and/or arterial hypertension as in our case^(3)^.

Herein, we present the first reported case of large preretinal hemorrhages that caused visual loss in the context of SARS-CoV-2 infection.
